# Augmented efficacy of intermittent theta burst stimulation on the virtual reality-based cycling training for upper limb function in patients with stroke: a double-blinded, randomized controlled trial

**DOI:** 10.1186/s12984-021-00885-5

**Published:** 2021-05-31

**Authors:** Yu-Hsin Chen, Chia-Ling Chen, Ying-Zu Huang, Hsieh-Ching Chen, Chung-Yao Chen, Ching-Yi Wu, Keh-chung Lin

**Affiliations:** 1 Department of Medicine, College of Medicine, Chang Gung University, Taoyuan, Taiwan; 2 Department of Physical Medicine and Rehabilitation, Chang Gung Memorial Hospital, Linkou, Taiwan; 3 Graduate Institute of Early Intervention, Chang Gung University, Taoyuan, Taiwan; 4 Neuroscience Research Center and Department of Neurology, Chang Gung Memorial Hospital, Linkou, Taiwan; 5 Institute of Cognitive Neuroscience, National Central University, Taoyuan, Taiwan; 6 Department of Industrial and Management, National Taipei University of Technology, Taipei, Taiwan; 7 Department of Physical Medicine and Rehabilitation, Chang Gung Memorial Hospital, Keelung, Taiwan; 8 Department of Occupational Therapy, College of Medicine, Chang Gung University, Taoyuan, Taiwan; 9 School of Occupational Therapy, College of Medicine, National Taiwan University, Taipei, Taiwan; 10 Division of Occupational Therapy, Department of Physical Medicine and Rehabilitation, National Taiwan University Hospital, Taipei, Taiwan

**Keywords:** Theta burst stimulation, Virtual reality, Stroke, Upper limb, Motor function, Rehabilitation

## Abstract

**Background:**

Virtual reality and arm cycling have been reported as effective treatments for improving upper limb motor recovery in patients with stroke. Intermittent theta burst stimulation (iTBS) can increase ipsilesional cortical excitability, and has been increasingly used in patients with stroke. However, few studies examined the augmented effect of iTBS on neurorehabilitation program. In this study, we investigated the augmented effect of iTBS on virtual reality-based cycling training (VCT) for upper limb function in patients with stroke.

**Methods:**

In this randomized controlled trial, 23 patients with stroke were recruited. Each patient received either 15 sessions of iTBS or sham stimulation in addition to VCT on the same day. Outcome measures were assessed before and after the intervention. Primary outcome measures for the improvement of upper limb motor function and spasticity were Fugl-Meyer Assessment-Upper Extremity (FMA-UE) and Modified Ashworth Scale Upper-Extremity (MAS-UE). Secondary outcome measures for activity and participation were Action Research Arm Test (ARAT), Nine Hole Peg Test (NHPT), Box and Block Test (BBT) and Motor Activity Log (MAL), and Stroke Impact Scale (SIS). Wilcoxon signed-rank tests were performed to evaluate the effectiveness after the intervention and Mann–Whitney U tests were conducted to compare the therapeutic effects between two groups.

**Results:**

At post-treatment, both groups showed significant improvement in FMA-UE and ARAT, while only the iTBS + VCT group demonstrated significant improvement in MAS-UE, BBT, NHPT, MAL and SIS. The Mann–Whitney U tests revealed that the iTBS + VCT group has presented greater improvement than the sham group significantly in MAS-UE, MAL-AOU and SIS. However, there were no significant differences in the changes of the FMA-UE, ARAT, BBT, NHPT and MAL-QOM between groups.

**Conclusions:**

Intermittent TBS showed augmented efficacy on VCT for reducing spasticity, increasing actual use of the affected upper limb, and improving participation in daily life in stroke patients. This study provided an integrated innovative intervention, which may be a promising therapy to improve upper limb function recovery in stroke rehabilitation. However, this study has a small sample size, and thus a further larger-scale study is warranted to confirm the treatment efficacy.

*Trial registration* This trial was registered under ClinicalTrials.gov ID No. NCT03350087, retrospectively registered, on November 22, 2017

## Background

Stroke is a leading cause of upper limb (UL) motor impairments. UL impairment commonly persists after the acute phase, resulting in long-term disability and decreased health-related life quality [1]. Despite receiving traditional neurorehabilitation programs, 50–60% of post-stroke patients remained functional motor limitations at variable degrees [2]. Various interventions and rehabilitation protocols have been developed in recent decades to enhance motor recovery and improve the quality of life in post-stroke patients. These rehabilitation programs include constraint-induced movement therapy, mirror therapy, and virtual reality (VR). Interventions include non-invasive brain stimulation (NIBS) and laser therapy.

Holden et al. identified repetition, positive feedback and patient’s motivation as the three key elements for post-stroke patients to achieve optimal functional recovery [3]. Therefore, this study combines VR with arm cycling to attain those elements. With the advancement of technology, VR has been increasingly utilized to treat neurological disorders. VR provides real-time somatosensory feedback to enhance motor control and learning [4], and initiates motivation for patients to endure repeated practice. Additionally, arm cycling was selected for the current rehabilitation program because it involves repetitive movement of bilateral upper limbs. Previous studies have demonstrated that bilateral extremities training induces interhemispheric facilitation [5], and that a repetitive training program provides additional benefit for functional recovery of upper limbs [6, 7]. Besides, unilateral virtual reality-based cycling training (VCT) was difficult for patients with hemiplegia. Taken together, this study applied bilateral VCT program for UL rehabilitation.

Repetitive transcranial magnetic stimulation (rTMS), a non-invasive brain stimulation technique, has been increasingly reported as a promising intervention that safely improves motor performance in the affected UL of stroke patients. Although the precise underlying mechanism remains unclear, rTMS is generally considered effective in improving functional outcome in patients with stroke by modulating motor cortical excitability and inducing reorganization of neural networks [8]. Since rTMS provides an environment to enhance neuroplasticity instead of skill acquisition, previous studies indicated combination therapy with rTMS and rehabilitative training improve motor functions to an extent that could not be attained by rTMS alone [9, 10]. For this reason, rTMS is often combined with motor behavioral intervention to enhance motor function. Intermittent theta burst stimulation (iTBS) is a variant of rTMS that may provide equivalent or even better efficacy. Therefore, this study explores the augmented efficacy provided by iTBS on the neurorehabilitation program to improve UL function.

Theta burst stimulation (TBS) is a novel stimulation protocol of rTMS that requires a lower stimulation intensity within a shorter time to achieve therapeutic effect in post-stroke patients [11]. Previous studies have indicated that TBS evoked comparable or even greater motor-evoked potentials (MEPs) [12] with longer-lasting effects than conventional rTMS methods [11]. Di Pino et al. proposed the bimodal balance-recovery model, integrating the interhemispheric competition and vicariation effect over the intact hemisphere, and suggested that stimulation protocol should be individualized according to the structural reserve [13]. The interhemispheric competition model was thought to predict recovery better in post-stroke patients with high structural reserve, while the vicariation theory is more relevant in post-stroke patients with low structural reserve. However, due to variable extent of residual neuronal networks, iTBS is generally applied to the ipsilesional primary motor cortex to facilitate cortical excitability, while continuous TBS (cTBS) is used to suppress the cortical excitability of the contralesional site based on the interhemispheric competition model [11]. The interhemispheric competition model indicates that cortical excitability decreases in the affected hemisphere following stroke, while transcallosal inhibitory signals from the unaffected hemisphere increase due to cortical hyperexcitability [14]. The increased cortical excitability in the intact hemisphere results in suppression of the ipsilesional hemisphere, which further leads to poor motor recovery in post-stroke patients [15]. Ward et al. found that the interhemispheric inhibition decreases with time, suggesting that cTBS has limited effect in stroke patients during chronic stage. Additionally, a recent meta-analysis revealed that iTBS has a better effect than cTBS for UL motor recovery in patients with stroke [16]. Therefore, iTBS was administered over the primary motor cortex of the ipsilesional hemisphere to assess its efficacy for improving UL function.

VCT aims to target the peripheral mechanisms of stroke recovery, while iTBS aims toward the central mechanisms by modulating cortical excitability [8]. Virtual reality also targets the central mechanisms by inducing cortical reorganization [17], which may cause a synergistic effect when combined with iTBS. A previous study revealed that combining low-frequency rTMS with VR training could improve UL function and quality of life in patients with subacute stroke [18]. Therefore, this study added iTBS on VCT to examine whether combining these two neurotechnologies shows additive effects, and whether central stimulation augments the effect of peripheral training.

To the best of our knowledge, this is the first randomized controlled trial to propose an innovative protocol adding iTBS on VCT, and to investigate the augmented efficacy of iTBS on VCT for upper limb motor function in patients with stroke. A 15-day intervention was implemented. Based on previous researches, iTBS was reported to reduce spasticity [19, 20] and improve motor function [19, 21]. We hypothesized that post-stroke patients completing a 15-day treatment program with iTBS and VCT have better UL function than the patients receiving sham stimulation and VCT.

## Methods

### Participants

Patients with stroke were recruited from the rehabilitation ward of Chang Kung Memorial Hospital. Inclusion criteria were: (1) first ever cerebral stroke; (2) under stable condition; (3) unilateral hemiplegia or hemiparesis due to unilateral cerebral stroke; (4) Brunnström stage of the affected upper limb ≥ 3, and (5) 30 to 70 years of age. Exclusion criteria were: (1) brainstem or cerebellar stroke; (2) history of seizure, brain aneurysm or arteriovenous malformation; (3) active psychiatric disease; (4) progressive neurodegenerative disease impairing cognitive function; (5) communicated disorders such as aphasia; (6) severe or active medical problems such as cardiac disease or pneumonia; (7) heavy metal implant; (8) pregnancy, (9) severe visual impairment; and (10) inability to follow instructions. All participants had signed the informed consent. The study was approved by Chang Gung medical foundation institutional review board and was registered under ClinicalTrials.gov ID No. NCT03350087.

### Design and experimental procedure

**Fig. 1:**
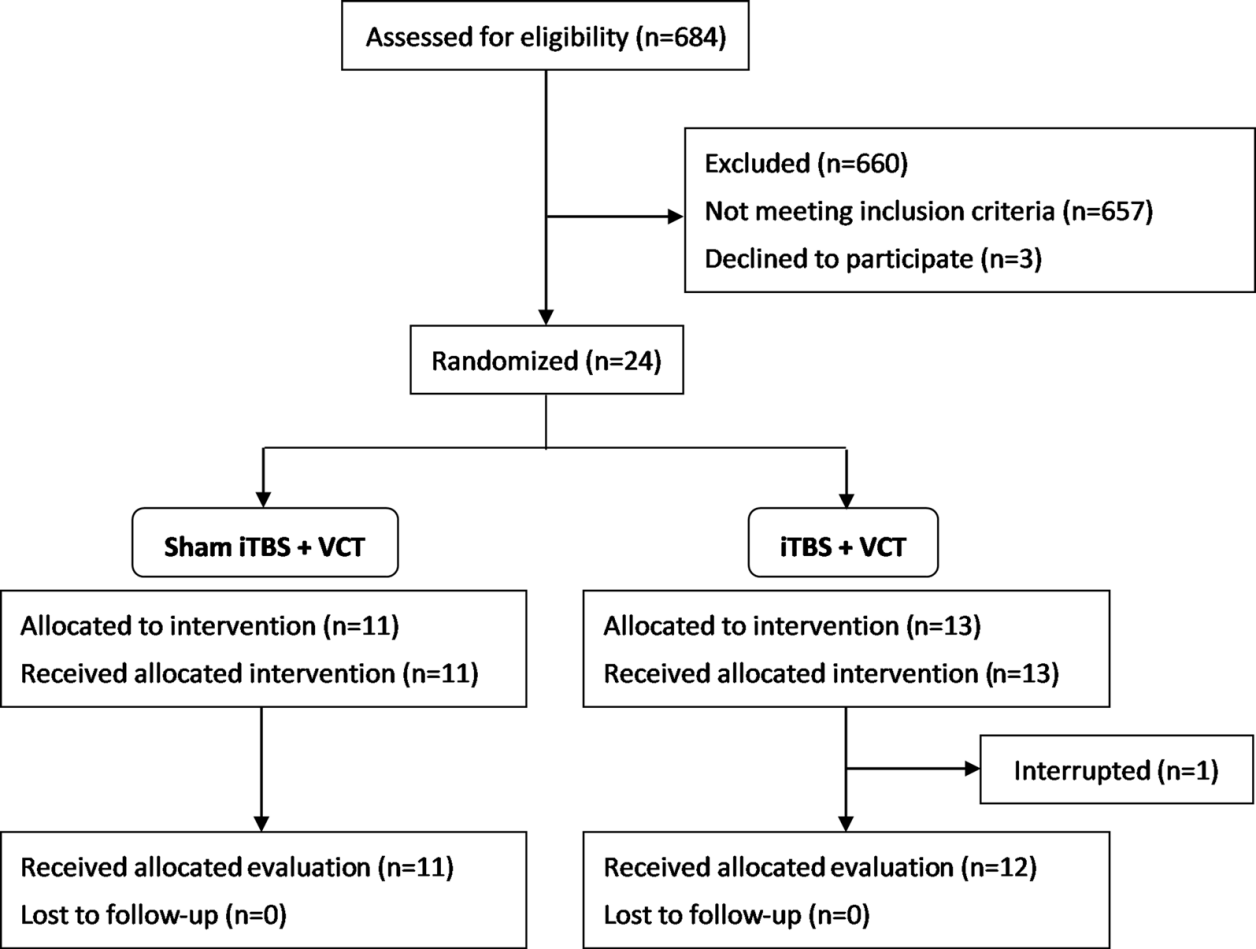
Flow diagram of recruitment and randomized allocation

**Fig. 2:**
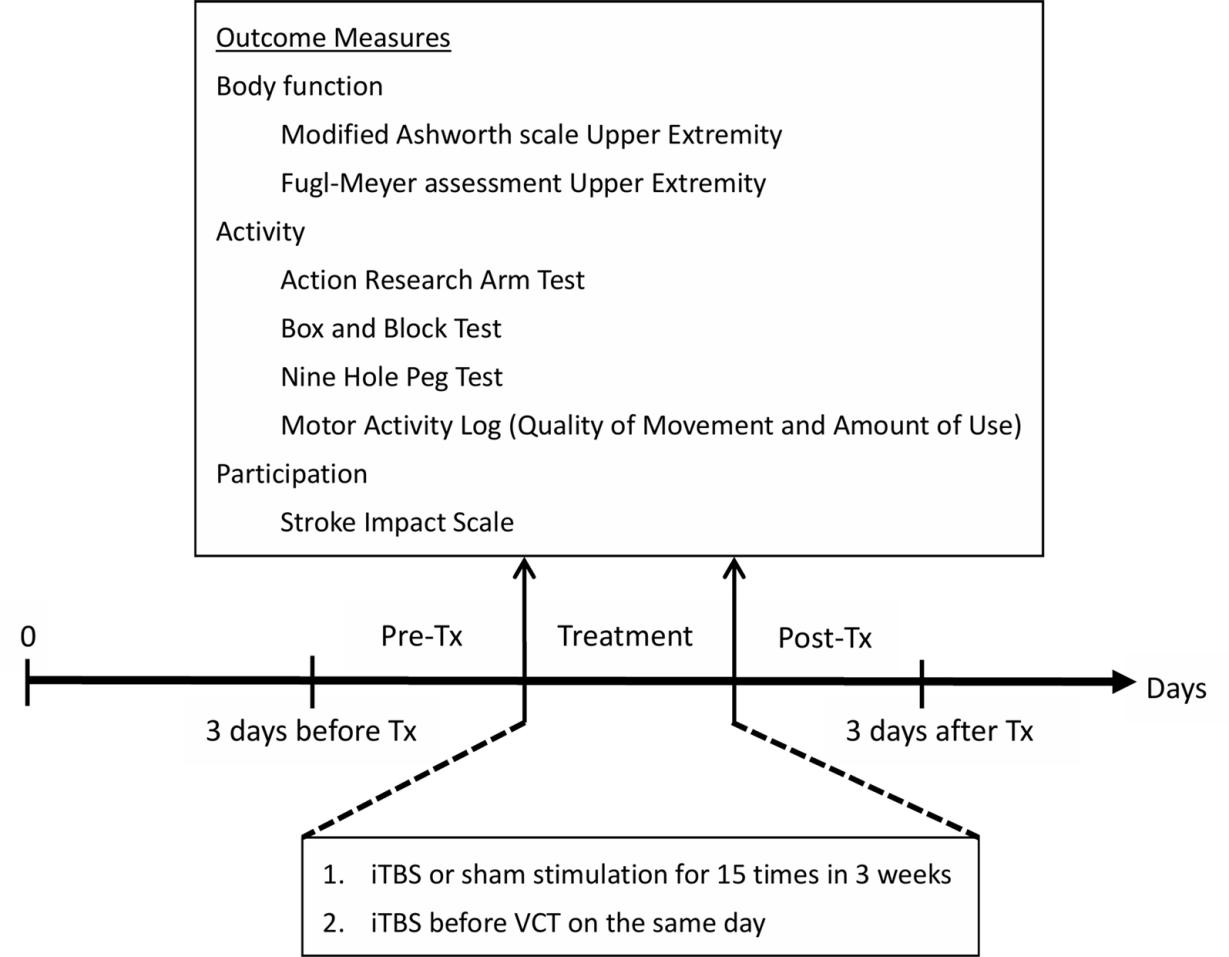
Experimental protocol

This study was a prospective, double-blinded and randomized controlled trial. Patients were randomly assigned to iTBS or sham stimulation in addition to VCT and were blind to the type of stimulation delivered. Randomized allocation was performed by generating a random sequence on the (https://www.randomizer.org/) website. Figures 1 and 2 illustrate schematic overviews of the randomized allocation and experimental procedure, respectively. Each patient received iTBS or sham stimulation before the 60-min VCT program on the same day for 15 consecutive working days (3 weeks). To avoid the contamination of physical activities on the effects of TBS, patients were told to avoid any movement of the affected upper limb 5 min before, during, and 5 min after the stimulation. We tried to avoid subjects’ physical activities and consolidate the effects of TBS in the period between TBS and VCT. Patients were then moved from the site of TBS stimulation to that of VCT by a wheelchair, and the distance between two sites was around 2 m. The training of VCT program was started as early as the setting of VCT was completed and the vital signs of patients were checked. In general, it took around 10 min between the end of TBS and the beginning of training. Patients were evaluated within 3 days before and after completing the therapy. Stimulation was conducted by the trained researchers, who were different from the raters. The outcome measures were administered by raters, occupational therapists, who contacted patients only during assessment and were blind to group assignment. The raters were trained before the experiment and evaluated by the written exam and reliability test. A 10-patient reliability test, measuring both intra-rater and inter-rater reliability, was conducted at 7-day intervals. The intra-rater/inter-rater reliability of the MAS-UE, FMA-UE, BBT and ARAT were analyzed by intra-class correlation as 0.841/0.841, 0.984/0.992, 1.000/0.998, and 0.986/0.998.

### Virtual reality-based cycling training

**Fig. 3:**
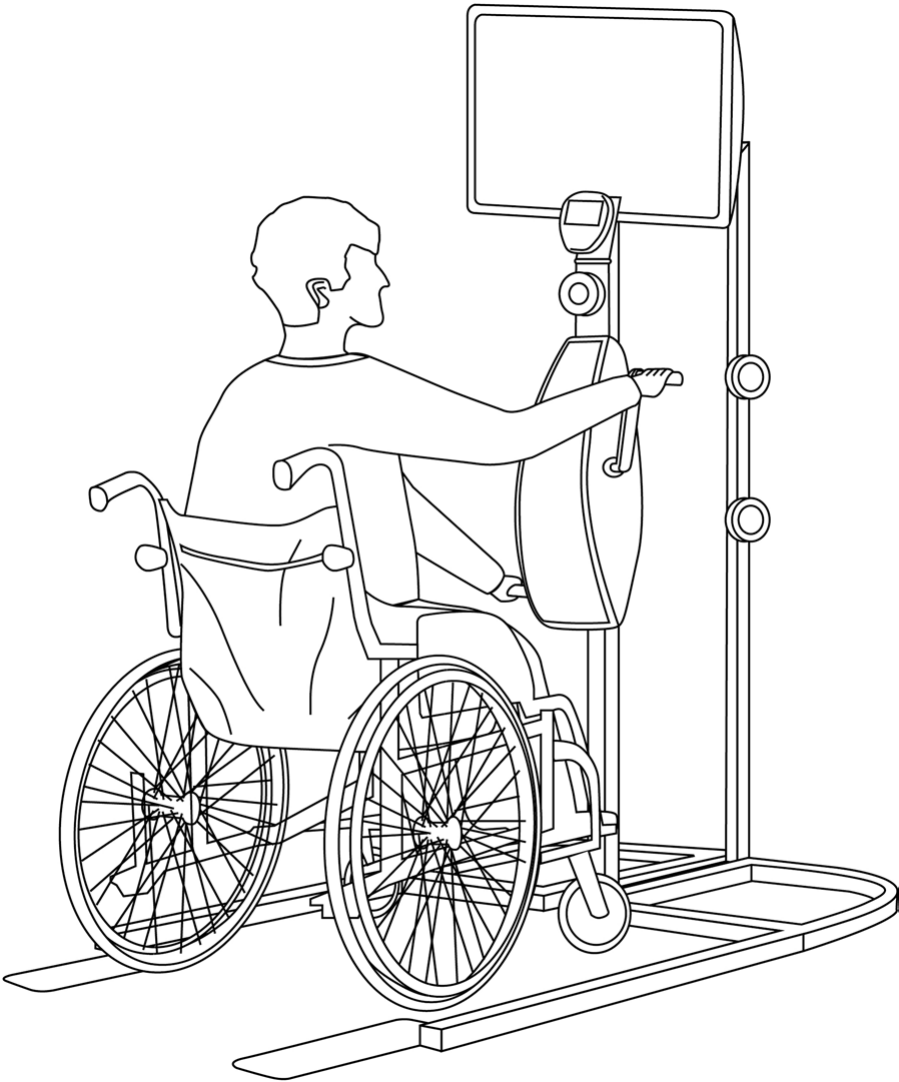
The setup of the VCT

This is the first study to administer VCT program over upper limbs in post-stroke patients, however, there were several studies regarding VCT program over lower limbs in stroke and other neurological disorders. VCT was reported to increase muscle strength of lower limbs in children with cerebral palsy [22] and to improve static balance in post-stroke patients [23]. Besides, a randomized controlled trial with 21 chronic patients found that bilateral arm training with rhythmic auditory cueing (BATRAC), a repetitive bilateral training therapy, induces reorganization in bilateral hemispheres [24]. For these reasons, VCT program for bilateral upper limbs rehabilitation was applied in the study. The VCT program comprised a warm-up exercise for 5 min, a 10-min weight training for upper limb including muscle strengthening, a 40-min cycling program composed of warm up, strength, and endurance training, and a 5-min cool down exercise. Dr. Hsieh-Ching Chen integrated virtual reality program with arm cycle (BK0010, X-BIKE Fitness Technology Company Limited) to comprise the virtual reality-based cycling system. The setup of the VCT was demonstrated in Fig. 3. During the training of the VCT program, patients would see themselves controlling the handlebar of a bicycle while riding on the road in different types of sceneries. The visual speed of the virtual scene was altered according to the signal of the cycling speed transmitted to the computer, increasing participants’ interest and motivation. Participants underwent low to moderate resistance and high revolutions per minute cycling exercise during the VCT program. Participants were encouraged to raise rpm during the program, aiming for the target heart rate based on the Karvonen Formula [25]. Thus, to ensure that participants achieve the target heart rate, the resistance was adjusted according to each participant’s clinical condition. To ensure participants’ safety, blood pressure, heart rate and oxygen saturation were monitored throughout the whole training program.

### Intermittent theta burst stimulation paradigm

iTBS was delivered over the hand motor area of the affected hemisphere by a handheld 70-mm standard, figure-of-eight coil connected to a MagPro X100 package (Magventure, USA). The optimal coil positioning over the scalp region was the motor hot spot, where the transcranial magnetic stimulation (TMS) evoked the largest MEP in the contralateral first dorsal interosseous (FDI) muscle with the patient at rest. Active motor threshold (AMT) was measured before each intervention, and was defined as the minimum TMS intensity required to evoke MEPs (≥ 200 μV) in at least 5 of 10 successive trials from the slightly contracted (approximately 10–20% of maximal strength) FDI muscle. True stimulation was applied over the identified motor hot spot at an intensity of 80% AMT, with the coil placed tangentially to the skull, at a 45° angle to the midsagittal axis, generating posterior–anterior current flow at targeted area in the brain. If MEPs could not be elicited, stimulation was applied to the mirror site of the motor hot spot over the unaffected hemisphere, as previous studies [19, 20], at the highest intensity that the stimulator could generate for TBS. Sham stimulation was administered at the same site with identical flip coil, resulting in a 78% output elicited by non-flip side, at a lower intensity (60% AMT) equivalent to 46.8% AMT [26]. The sham stimulation with intensity lower than 70% AMT has no effect on MEPs, as demonstrated by a previous study [27], but produces indistinguishable sensation and sound compared to real stimulation. Several previous studies administered a similar sham stimulation method, and found it to be useful [19, 26, 28]. All patients were seated comfortably with their hands as relaxed as possible throughout the experiment after the AMT was recorded. An iTBS session comprised 2-s train of bursts, containing three pulses at 50 Hz, repeated at intervals of 200 ms, every 10 s for 20 times (a total of 600 pulses). Learning from studies on spaced TBS [29, 30], two sessions of iTBS were applied with a 10-to-15-min break for a total of 1200 pulses to consolidate and induce longer-lasting changes in cortical excitability. Therefore, two sessions of iTBS with 600 pulses with a 10-min break to have 1200 pulses in total were given to enhance the modulation effects. Patients were instructed to rest and not to move during the 10-min break in order to minimize cofounding factors and not to disrupt any ongoing plasticity [31]. Real or sham iTBS was delivered for 15 consecutive work days.

### Outcome measures

Primary outcome measures were the improvement of upper limb motor function and spasticity, measuring by FMA-UE and MAS-UE, respectively. FMA-UE is a performance-based scale, particularly for patients with stroke, to assess sensorimotor function including motor function, joint function, sensation and balance [32]. This study only evaluated the UL motor function of FMA. MAS-UE, which is scored from 0 to 4 (0, 1, 1+, 2, 3, 4), was used to assess UL spasticity and resistance during passive joint movement [33, 34]. The affected finger flexor muscles, wrist and elbow were evaluated. These MAS-UE scores were summed to represent the UL spasticity, with 1+ calculated as 1.5.

Secondary outcome measures were the domains of activities and participation. The improvement of activity was evaluated using ARAT, BBT, NHPT and MAL. ARAT measures UL motor function, and comprises 19 items divided into four subsets: grasp, grip, pinch, and gross movement (GM) [35]. BBT is a functional test to measure unilateral gross manual dexterity [36], in which patients have to move as many blocks from one box to another box as possible in 60 s only by the affected hand, the task requires grasping, transporting and releasing. NHPT is a timed test performed to evaluate manual dexterous function [37], in which patients insert nine pegs into nine holes of the pegboard and then pick them up as quickly as possible. Since the patients with severe motor impairment might be unable or needed a long time to complete the task, patients were asked to perform NHPT within 2 min. To distinguish the ability of the patients who could not complete the task in 2 min, the number of pegs being placed and removed was calculated. The outcome variable is the number of pegs/minute, with more pegs/minute indicating better dexterity. MAL was assessed to determine patients’ real life functional performance involving the affected arm based on 14 daily activities [38], including amount of use and quality of movement.

SIS, a patient-reported questionnaire, was performed to evaluate participation in patients with stroke [39]. SIS is a measure specific to patients with stroke, with higher scores reflecting greater participation. The outcome variable is presented as the average score of the eight domains.

### Statistical analysis

All statistical analyses were conducted with SPSS version 21 (SPSS Inc., Chicago, Illinois). Shapiro–Wilk tests were conducted to confirm assumptions of normality of distributions. However, only the data of MAS-UE, FMA-UE, and SIS follow the normal distribution. Therefore, under non-normal distribution and small sample size, nonparametric methods were used in the current study. To determine the baseline between-group differences of demographic characteristics, Chi-square tests were applied for the categorical variables and Mann–Whitney U tests were conducted for the continuous variables. In order to assess whether the iTBS + VCT group had greater therapeutic effect than the sham iTBS + VCT group, Mann–Whitney U tests were applied to compare the change scores (posttest scores − pretest scores) between groups. Mann–Whitney U tests were also applied to compare the changes of the stimulated intensity (motor threshold) between groups. Wilcoxon signed-rank tests were run to test whether each group showed significant improvement after the therapy. Statistical significance level was set at *p* < 0.05 (one-tailed) [40] for all analyses of treatment effects under directional hypothesis [41]. Under a small sample size (n < 30), T-distribution was used to compute a 95% confidence interval (95% CI).

## Results

A total of 684 patients were screened, among whom 657 patients were excluded and three patients declined to participate. Twenty-four patients were randomly allocated to the iTBS + VCT or the sham iTBS + VCT group, and one patient in the iTBS + VCT group withdrew from the study. Ultimately, 12 patients in the iTBS + VCT group and 11 patients in the sham iTBS + VCT group completed the study course. The time since stroke onset of all the patients was greater than 3 weeks. Nine patients were diagnosed as stroke with subcortical lesion in each group, whereas three patients in iTBS + VCT group and two patients in sham iTBS + VCT group had cortical lesions. Among five patients with cortical lesions, two patients in each group had M1 involvement. The demographic and clinical data did not differ between two groups (Table 1). Although the NIHSS in the sham iTBS + VCT group is greater than the iTBS + VCT group, it did not reach significant differences. There were four patients with no measurable MEP in each group. All patients could tolerate the intervention without significant adverse effects throughout the study. Throughout the treatment course, only one patient mentioned upper limbs muscle soreness after receiving VCT training program. The discomforts relieved after taking rest and ice packing. No significant baseline between-group differences in outcome measures were observed (Table 2).

**Table 1:** Demographic and clinical characteristics

	sham iTBS + VCT	iTBS + VCT	*p*-value
Age	48.95 ± 9.63	54.36 ± 10.56	0.316^a^
Gender			0.317^b^
Male	10 (90.9%)	8 (66.7%)	
Female	1 (9.1%)	4 (33.3%)	
Onset time (month)	7.99 ± 5.41	5.01 ± 4.39	0.449^a^
Stroke type			0.193^b^
Infarction	2 (18.2%)	6 (50.0%)	
Hemorrhage	9 (81.8%)	6 (50.0%)	
Stroke side			1.000^b^
Right	4 (36.4%)	5 (41.7%)	
Left	7 (63.6%)	7 (58.3%)	
Stroke location			1.000^b^
Cortical	2 (18.2%)	3 (25.0%)	
M1 involvement	2 (18.2%)	2 (16.7%)	
Subcortical	9 (81.8%)	9 (75.0%)	
MEP			0.879^b^
Positive	7 (63.6%)	8 (66.7%)	
Negative	4 (36.4%)	4 (33.3%)	
Aphasia			0.640^b^
Yes	3 (27.3%)	2 (16.7%)	
No	8 (72.7%)	10 (83.3%)	
NIHSS	13.55 ± 2.38	11.92 ± 1.73	0.190^a^

Data are presented as mean ± standard deviation or number (%)

*iTBS* intermittent theta burst stimulation, *MCA* middle cerebral artery, *MEP* motor evoked potential, *NIHSS* National Institutes of Health Stroke Scale

^a^Mann-Whitney U tests

^b^Chi-square tests

**Table 2:** Baseline of outcome measures

	sham iTBS + VCT	iTBS + VCT	*p*-value
FMA-UE	34.55 ± 18.34	43.58 ± 15.35	0.288
MAS-UE	0.94 ± 0.69	0.87 ± 0.54	0.786
ARAT	17.09 ± 18.11	25.75 ± 22.69	0.487
GM	4.73 ± 1.68	5.33 ± 2.87	0.608
Grasp	5.55 ± 6.65	8.75 ± 8.01	0.288
Grip	3.00 ± 4.31	5.42 ± 5.16	0.379
Pinch	3.82 ± 6.31	6.25 ± 7.40	0.379
BBT	11.40 ± 16.02	18.72 ± 18.84	0.379
NHPT^a^	4.02 ± 8.82	7.86 ± 11.88	0.413
MAL (AOU)	42.64 ± 31.04	33.92 ± 42.40	0.316
MAL (QOM)	46.55 ± 40.89	35.17 ± 42.58	0.379
SIS	57.09 ± 8.61	58.06 ± 12.79	0.833

Data are presented as mean ± standard deviation

*iTBS* intermittent theta burst stimulation, *VCT* virtual reality-based cycling training, *MAS-UE* Modified Ashworth Scale-Upper Extremity, *FMA-UE* Fugl-Meyer Assessment-Upper Extremity, *ARAT* Action Research Arm Test, *GM* gross movement, *BBT* Box and Block Test, *NHPT* Nine Hole Peg Test, *MAL(AOU)* Motor Activity Log (Amount of Use), *MAL (QOM)* Motor Activity Log (Quality of Movement), *SIS* Stroke Impact Scale

^a^The unit of NHPT is number of pegs/minute

### Primary outcomes

After the intervention, both groups showed significant improvement in FMA-UE (control: *p* = 0.003; iTBS: *p* = 0.021), while only the iTBS + VCT group showed significant improvement in MAS-UE (control: *p* = 0.336; iTBS: *p* = 0.004). Mann–Whitney U tests revealed that the iTBS + VCT group induced significantly greater gains than the control group in MAS-UE (*p* = 0.007) after the intervention, while there was no significant difference in score change between two groups in FMA-UE (*p* = 0.174) (Table 3). Seven patients in iTBS + VCT group reached the minimal clinically important difference (MCID) of MAS-UE, whereas only one patient reached the MCID of MAS-UE (− 0.19) [42] in sham iTBC + VCT group. In FMA-UE, two patients in iTBS + VCT group and three patients in sham iTBS + VCT group reached MCID (9 points) [43].

**Table 3:** Descriptive and inferential statistics of outcome measures

Variables	sham iTBS + VCT (Wilcoxon signed-rank tests)					iTBS + VCT (Wilcoxon signed-rank tests)					M–W U tests
	Pre-Tx		Post-Tx		*p*	Pre-Tx		Post-Tx		*p*	*p*
	Mean ± SD	95% CI	Mean ± SD	95% CI		Mean ± SD	95% CI	Mean ± SD	95% CI		
FMA-UE	34.55 ± 18.34	23.05–46.05	40.64 ± 16.83	30.08–51.19	0.003^†^	43.58 ± 15.35	34.36–52.80	47.17 ± 16.30	37.38–56.96	0.021*	0.174
MAS-UE	0.94 ± 0.69	0.51–1.38	0.97 ± 0.63	0.57–1.36	0.336	0.87 ± 0.54	0.54–1.19	0.65 ± 0.50	0.34–0.95	0.004^†^	0.007^†^
ARAT	17.09 ± 18.11	5.73–28.45	18.27 ± 18.91	6.42–30.13	0.027*	25.75 ± 22.69	12.13–39.37	30.42 ± 22.38	16.98–43.85	0.025*	0.225
GM	4.73 ± 1.68	3.67–5.78	5.36 ± 1.80	4.23–6.50	0.010*	5.33 ± 2.87	3.61–7.06	6.50 ± 2.88	4.77–8.23	0.006^†^	0.158
Grasp	5.55 ± 6.65	1.37–9.72	5.27 ± 6.77	1.03–9.52	0.090	8.75 ± 8.01	3.94–13.56	9.50 ± 7.74	4.85–14.15	0.207	0.190
Grip	3.00 ± 4.31	0.30–5.70	3.55 ± 4.55	0.69–6.40	0.090	5.42 ± 5.16	2.32–8.52	6.83 ± 5.38	3.61–10.06	0.390	0.326
Pinch	3.82 ± 6.31	-0.14–7.77	4.09 ± 6.33	0.12–8.06	0.159	6.25 ± 7.40	1.81–10.69	7.58 ± 7.56	3.04–12.12	0.055	0.263
BBT	11.40 ± 16.02	1.20–21.60	11.88 ± 13.74	3.00–20.40	0.393	18.72 ± 18.84	7.20–30.00	21.96 ± 19.50	10.20–33.60	0.029*	0.130
NHPT^a^	4.02 ± 8.82	-1.20–9.60	4.56 ± 7.92	-0.60–9.60	0.208	7.86 ± 11.88	0.60–15.00	11.40 ± 13.86	3.00–19.80	0.023*	0.106
MAL (AOU)	42.64 ± 31.04	23.17–62.10	36.55 ± 22.06	22.71–50.38	0.118	33.92 ± 42.40	8.46–59.37	42.25 ± 43.93	15.87–68.63	0.009^†^	0.006^†^
MAL (QOM)	46.55 ± 40.89	20.90–72.19	42.82 ± 32.96	22.15–63.49	0.337	35.17 ± 42.58	9.60–60.73	42.83 ± 43.39	16.78–68.88	0.009^†^	0.076
SIS	57.09 ± 8.61	51.69–62.49	58.04 ± 6.47	53.99–62.10	0.066	58.06 ± 12.79	50.38–65.73	65.02 ± 13.50	56.91–73.12	0.001^†^	0.005^†^

T-distribution was used to compute a 95% CI. Degrees of freedom = 22

*SD* standard deviation, *CI* confidence interval, *M–W U tests* Mann–Whitney U tests, *iTBS* intermittent theta burst stimulation, *VCT* virtual reality-based cycling training, *MAS-UE* Modified Ashworth Scale-Upper Extremity, *FMA-UE* Fugl-Meyer Assessment-Upper Extremity, *ARAT* Action Research Arm Test, *GM* gross movement, *BBT* Box and Block Test, *NHPT* Nine Hole Peg Test, *MAL(AOU)* Motor Activity Log (Amount of Use), *MAL (QOM)* Motor Activity Log (Quality of Movement), *SIS* Stroke Impact Scale

*p < 0.05

^†^*p* < 0.01

^a^The unit of NHPT is number of pegs/minute

### Secondary outcomes

#### Activity

After the intervention, both groups showed significant improvement in ARAT (control: *p* = 0.027; iTBS: *p* = 0.025), while there was no significant difference in score change between two groups (*p* = 0.225). Among all the domains of ARAT, both groups showed significant improvement only in GM domain of ARAT (control: GM: *p* = 0.010, Grasp: *p* = 0.090, Grip: *p* = 0.090, Pinch: *p* = 0.159; iTBS: GM: *p* = 0.006, Grasp: *p* = 0.207, Grip: *p* = 0.390, Pinch: *p* = 0.055). Results of Mann–Whitney U tests revealed that all domains of ARAT did not differ between two groups (GM: *p* = 0.158; grasp: *p* = 0.190; grip: *p* = 0.326; pinch: *p* = 0.263). In ARAT, four patients in iTBS + VCT group and one patient in sham iTBS + VCT group reached MCID (5.7 points) [44].

In BBT, Wilcoxon signed-rank tests revealed that only the iTBS + VCT group had significant improvement after the intervention (control: *p* = 0.393; iTBS: *p* = 0.029), and Mann–Whitney U tests showed that the iTBS + VCT group had no greater gains than the sham iTBS + VCT group (*p* = 0.130). In NHPT, Wilcoxon signed-rank tests revealed that only the iTBS + VCT group had significant improvement after the intervention (control: *p* = 0.208; iTBS: *p* = 0.023), and Mann–Whitney U tests showed no greater gains in the iTBS + VCT group than the sham iTBS + VCT group (*p* = 0.106). In BBT, four patients in iTBS + VCT group and two patients in sham iTBS + VCT group reached MCID (six blocks) [45]. There was no reported MCID of NHPT under the unit used in the current study.

In MAL, the iTBS + VCT group showed significant improvement after the intervention, and the sham iTBS + VCT group had no significant improvement in MAL (control: MAL-AOU: *p* = 0.118, MAL-QOM: *p* = 0.337; iTBS: MAL-AOU: *p* = 0.009, MAL-QOM: *p* = 0.009). Mann–Whitney U tests revealed significant between-group differences in the gains following the intervention only in MAL-AOU (MAL-AOU: *p* = 0.006; MAL-QOM: *p* = 0.076). In both MAL-AOU and MAL-QOM, two patients in iTBS + VCT group and none of the patients in sham iTBS + VCT group reached minimal detectable change 90 (MDC_90_) (MAL-AOU: 0.84; MAL-QOM: 0.77) [46].

#### Participation

In SIS, Wilcoxon signed-rank tests showed that only the iTBS + VCT group had significant improvement after the intervention (control: *p* = 0.066; iTBS: *p* = 0.001), and Mann–Whitney U tests revealed that iTBS + VCT group had greater gains than the sham iTBS + VCT group (*p* = 0.005). In SIS, two patients in iTBS + VCT group and none of the patients in sham iTBS + VCT group reached MCID, which was defined as 10% of the total scale [32].

## Discussion

To the best of our knowledge, this is the first exploratory trial to test whether TBS had augmented efficacy on VCT for upper limb function. In the current study, iTBS induced significantly greater gains in the MAS-UE, MAL-AOU and SIS than sham stimulation. However, the changes in FMA-UE, ARAT, BBT, NHPT and MAL-QOM did not differ between the two groups. These findings indicate that iTBS augments the effect of VCT on reducing spasticity, increasing actual use of the affected UL, and improving participation. Since this is the first study to perform iTBS on VCT in stroke patients, our findings were compared with those of studies adding iTBS on other neurorehabilitation program.

There are several factors that may influence the interpretation of the results, including the time since stroke, stroke types and stroke locations. The time since stroke between the two groups showed no significant difference, although the average time since stroke in the iTBS + VCT group was shorter than the sham iTBS + VCT group. Several studies demonstrated increased neuroplasticity and greater behavioral recovery at the early stage after stroke [47, 48], which may take into consideration while interpreting the results. The major stroke type in our study was hemorrhagic stroke despite approximately 87% of stroke are ischemic [49]. One explanation is that hemorrhagic stroke is generally more severe compared to ischemic stroke, and is often transferred to rehabilitation ward after stable condition. In this study, participants were recruited from the rehabilitation ward instead of outpatient clinic. Most of the patients with stroke in the study had subcortical lesions. Among these patients, two patients in each group had M1 involvement, which was the target area for the stimulation. Although the impact of TBS was not only local, the beneficial effect may differ according to different stroke locations [50].

Experimental results revealed that the iTBS + VCT group showed greater reduction in spasticity than the sham iTBS + VCT group in stroke patients. Our findings were consistent with those of a randomized controlled trial indicating that iTBS showed a significant reduction of spasticity in patients with chronic stroke [19], and were also compatible with another study demonstrating that a single session of iTBS significantly reduced UL spasticity transiently in patients with acute and chronic stroke [20]. The minimal clinically important differences (MCID) of MAS of large and medium effect size were reported to be 0.76 and 0.48, respectively [42]. Based on the equation provided in the previous study [42], the MCID of MAS of small effect size (0.2 standard deviations) was 0.19. Despite the mean improvement after receiving iTBS and VCT in the study was 0.22, which did not meet the medium effect size, it reached a small effect size. Overall, iTBS showed augmented effect on VCT for reducing spasticity in stroke patients.

Spasticity is a common cause of long-term disability in stroke patients. The postulated pathophysiology of spasticity is that lesions of upper motor neuron impair the supraspinal inhibitory inputs, leading to an increased excitability of α and γ motor neurons, and of the interneurons at the spinal level [51, 52]. Therefore, facilitatory rTMS and iTBS have been applied to reduce spasticity in patients with a number of neurologic disorders [19, 20, 53–58]. It is increasingly accepted that iTBS may induce the long-term potential-like (LTP-like) plasticity changes [11, 59, 60], and may further project to inhibitory corticospinal synapses. Additionally, iTBS may also alter the level of endogenous transmitters involving in synaptic plasticity [57, 61] such as γ-aminobutyric acid [62], glutamate [63] and dopamine [64]. The mechanism for the anti-spastic effect of iTBS remains unclear to date, and further neurophysiological studies are warranted to identify the underlying mechanism.

In the current study, both groups showed significant improvement in FMA after the intervention, but the changes after the intervention revealed no significant differences between the iTBS + VCT and sham iTBS + VCT group. One possible explanation is that virtual reality (VR) itself generates an enriched environment providing sensorimotor stimulation and leads to improvement in upper limb motor function [65–67]. Conversely, arm cycling involves repetitive bilateral arm training and is able to improve upper limb motor function [68]. Our findings were partially consistent with previous studies [19, 69]. Hsu et al. found that six patients with subacute ischemic stroke receiving iTBS had measurable improvement in the proximal UL motor function compared with the other six patients receiving sham stimulation [69]. Chen et al. revealed that iTBS had significant effect on upper limb motor function measured by the FMA in patients with chronic stroke [19]. Zheng et al. found that combining low-frequency rTMS and VR training showed prominent effects at the 2nd, 3rd, and 4th week after the intervention [18]. The application of iTBS over the ipsilesional hemisphere was based on the vicariation theory, proposing that surviving neurons situated at the peri-infarct area may be reorganized and substitute the function of the stroke region [70, 71]. Since the mechanisms of upper limb motor recovery included the vicarious capacity of the primary motor cortex (M1), facilitation of the affected hemisphere may arouse compensatory neural plasticity adjacent to the lesion and rebalance cortical excitability between hemispheres. Overall, our study revealed that iTBS may have no additionally augmented effect on VCT in UL impairment.

In the current study, only the iTBS + VCT group showed significant improvement after the intervention in both BBT and NHPT, while the sham iTBS + VCT group did not. However, iTBS + VCT induced no greater gains than sham iTBS + VCT in both BBT and NHPT. These results were not compatible with those of some previous studies [19, 21, 72]. Talelli et al. reported that six patients with chronic stroke had shorter simple reaction times of gripping tasks after iTBS than after sham stimulation [21]. Malcolm et al. demonstrated that the group receiving rTMS as an adjuvant therapy to constraint-induced therapy had greater gains than the sham stimulation group in BBT at 6 months [72]. A more recent study by Chen et al. reported that iTBS significantly improved the performance in BBT in 22 patients with chronic stroke [19]. These variable findings may be owing to different combined treatment protocols, different patient characteristics, since inter-individual response variability following iTBS had been observed [70]. Stimulation protocols, intensity and location may also influence the effect of rTMS on neural activity. In summary, our results indicate that iTBS combined with VCT may have the potential to improve the fine motor function. However, iTBS showed no additional benefit on VCT for the recovery of manual dexterity.

After the intervention, both groups showed significant improvement only in gross motor domain. Besides, the iTBS + VCT group showed no greater gains than the sham iTBS + VCT group. A previous study by Ackerley et al. found that iTBS priming with physical therapy, but not sham stimulation, enhanced the improvement in ARAT, and could be maintained for 1 month in 18 patients with chronic stroke [73]. Chen et al. reported that iTBS showed greater improvement than the control group in fine motor domains including pinch, grasp, and grip, but not in gross motor domain [19]. These findings could be explained by different neurorehabilitation protocols. Gross motor movement mainly involves shoulder and elbow, which were the major parts trained by VCT. Although there was no significant difference in the ARAT change between groups, patients receiving iTBS + VCT in our studies had significant improvement in the gross motor domain of ARAT as comparing to the baseline. In current study, treatment with iTBS + VCT might show the potential to have benefits on gross motor recovery, although iTBS had no augmented efficacy on VCT to improve all the domains of motor function in ARAT.

This study demonstrated that only the iTBS + VCT group showed significant improvement in MAL after the intervention. Furthermore, the changes in MAL-AOU after intervention achieve significant differences between two groups. However, previous studies showed that stimulation had no greater gains compared to the control group in both MAL-AOU and MAL-QOM [19, 72]. Malcolm et al. found that the changes after the intervention in both MAL-AOU and MAL-QOM did not differ between the ten sessions of rTMS and sham stimulation at time points of 2 weeks and 6 months [72]. In addition, Chen et al. also indicated that the iTBS group had no greater improvement than the sham group in MAL-AOU and MAL-QOM [19]. To explain current result, both bilateral repetitive movement and iTBS may play an important role. Bilateral symmetrical movement was reported to facilitate both hemispheres and to reduce intra-cortical inhibition [74]. Besides, iTBS was thought to activate the affected hemisphere and further suppress the unaffected hemisphere, thereby correcting imbalanced interhemispheric competition [75]. Furthermore, balanced interhemispheric interactions are reported to be necessary for normal voluntary movements [76], and this may explain the result. However, interhemispheric competition model is not the only proposed concept to explain the therapeutic effect of iTBS, and therefore the precise underlying mechanism remains unknown. Although combining iTBS with VCT might overcome the compensatory strategy after stroke, the improvement made by the stimulation was not sufficient to benefit quality of movement. To sum up, iTBS showed augmented efficacy on VCT in increasing actual use of the affected UL.

The present study revealed that only the iTBS + VCT group showed significant improvement in SIS after the intervention. Furthermore, the iTBS + VCT group had greater gains than the sham iTBS + VCT group. SIS comprises various aspects including strength, hand function, ADL/IADL, mobility, emotion, communication, memory and thinking, and participation. Based on previous study, SIS was recommended as an outcome measure to assess the improvement in participation after receiving a VR-based treatment [77]. To our best knowledge, this is the first study to assess SIS in patients with stroke after iTBS. However, our findings were not compatible with those of a previous study, which reported that rTMS as an adjuvant therapy to task-oriented training showed no greater gains than sham stimulation in SIS [78]. These variable findings may due to different protocols. To further explore the result, the therapeutic effect in different aspects of SIS was analyzed. The iTBS + VCT group had greater gains than the sham iTBS + VCT group in the aspects of mobility and participation. A previous study reported that arm cycling training improved walking ability and balance [79]. These findings may result from enhancing interlimb connectivity, reflex control, and locomotor central pattern-generating networks, controlling both arm cycling and walking [79]. Therefore, the positive effects of arm cycling may further improve the participation in the mobility domain. As for the aspect of participation, among the eight questions, active recreation, the role as a family member, and the ability to help others were self-reported to have greater gains in the iTBS group than the sham group. One explanation was that theta bust stimulation was reported as anti-depression treatment [80, 81], and recovering from post-stroke depression might enable the patients to communicate more effective with others. However, the stimulation site for depression is different from that for motor impairment. Therefore, further studies are warranted to identify the underlying mechanism. In conclusion, since this study found that iTBS can augment the effect of VCT on improving manual dexterity and reducing spasticity, iTBS can also be reasonably considered to augment the effect of VCT on enhancing participation.

MEP is known to represent the functional integrity of the descending corticospinal tract. The iTBS + VCT group showed functional improvement after receiving the intervention, although 33.3% of the patients in iTBS + VCT group had absence of MEP. Despite the absence of MEP in M1indicates severe damage of functional integrity in M1, the functional integrity in the other cortex may be reserved. Furthermore, previous studies reported that secondary motor networks are recruited to generate descending motor output when pathways from primary motor area (M1) are damaged [82, 83]. Besides, iTBS was found to have remote effects over the motor-related areas in the previous study [84]. These studies may explain our results. To further uncover the mechanism under the augmented effect of iTBS on VCT, Mann–Whitney U tests was conducted to compare the changes in the motor thresholds of MEPs between two groups. Although iTBS + VCT group showed more changes in decreased motor thresholds than the sham iTBS + VCT group, there was no significant between-group difference (*p* = 0.085).

This study has several limitations. First, convenience sampling was used to select participants from a rehabilitation ward because this is the first study to investigate the augmented efficacy of iTBS on the VCT for upper limb function in patients with stroke. A large-scale survey with a probability sampling strategy and a sample size more than 27 participants is warranted to generalize and confirm the clinical benefits. Second, no follow up for the long-term effect of iTBS was performed, hence further studies should also trace for the lasting efficacy. Third, the study recruited patients with age only between 30 and 70 years, and therefore the findings in the study may not extend to younger or older patients. Fourth, no multiple comparisons were conducted to control type II errors [85], considering the fact that this is a small sample-sized study and the nature of the study was to explore the efficacy of a novel intervention.

## Conclusion

Applying iTBS over the ipsilesional hemisphere had augmented efficacy on VCT in reducing spasticity, increasing actual use of the affected upper limb, and improving participation in daily life. Additionally, no patients experienced significant acute side effects after receiving iTBS in all patients. In conclusion, iTBS may be a promising and safe treatment option as an adjuvant therapy that could augment the therapeutic effects of neurorehabilitation in stroke patients. A further larger-scale study is warranted to verify the results.

## Data Availability

The datasets used and/or analyzed during the current study are available from the corresponding author on reasonable request.

## Abbreviations

ADL: Activities of daily living
AMT: Active motor threshold
AOU: Amount of use scale
ARAT: Action research arm test
BBT: Box and Block Test
cTBS: Continuous TBS
FMA-UE: Fugl-Meyer Assessment-Upper Extremity
iTBS: Intermittent TBS
M1: Primary motor cortex
MAL: Motor activity log
MAS-UE: Modified Ashworth scale-Upper Extremity
MEPs: Motor-evoked potentials
NHPT: Nine hole peg test
QOM: Quality of Movement scale
RCT: Randomized controlled trial
rTMS: Repetitive transcranial magnetic stimulation
SIS: Stroke Impact Scale
TBS: Theta burst stimulation
UL: Upper limb
VCT: Virtual reality-based cycling training
VR: Virtual reality
